# Supplementary material for: Urinary Potassium Excretion, Fibroblast Growth Factor 23, and Incident Hypertension in the General Population-Based PREVEND Cohort
Source: Nutrients. 2021 Dec 17;13(12):4532. doi: 10.3390/nu13124532 (PMC8707837; doi:10.3390/nu13124532)
Supplement: Supplementary file 1 [file nutrients-13-04532-s001.zip › nutrients-1515098-supplementary.pdf]

Supplemental material

Supplemental Table S1. Baseline characteristics according to sex specific tertiles of fibroblast growth factor 23 in 4,178 participants of PREVEND

	Sex-specific tertiles of total fibroblast growth factor 23, RU/mL			
	**I**	**II**	**III**	***p*-trend***
Men	<58	58–73	≥73	
Women	<61	61–82	≥82	
Fibroblast growth factor 23, (RU/mL)	49 (44 – 53)	61 (58 – 64)	74 (70 – 79)	-
Men, n (%)	648 (46)	649 (46)	646 (46)	-
Age, (yr.)	48 (10)	50 (10)	50 ± 11	<0.001
BMI, (kg/m^2)	24.9 (23.0 – 27.4)	25.3 (23.1 – 27.9)	25.6 (23.2 – 28.4)	<0.001
Systolic blood pressure, (mmHg)	117 (109 – 126)	117 (109 – 126)	118 (110 – 127)	0.07
Diastolic blood pressure, (mmHg)	70 (65 – 76)	70 (65 – 75)	70 (66 – 76)	0.14
Lipid lowering drugs, n (%)	32 (3)	41 (3)	56 (5)	0.05
Antidiabetic drugs, n (%)	13 (1)	21 (2)	20 (2)	0.42
Smoking status				<0.001
Never, n (%)	495 (36)	420 (30)	375 (27)	
Current or former, n (%)	897 (64)	976 (70)	1015 (73)	
Alcohol consumption, yes (%)	1114 (80)	1084 (78)	1036 (74)	0.003
Cardiovascular disease, yes (%)	57 (6)	71 (8)	55 (6)	0.20
Diabetes, yes (%)	30 (2)	42 (3)	46 (3)	0.17
eGFR (CKD-epi), (mL/min·1.73m^2)	99 (13)	96 (13)	95 (14)	<0.001
Plasma albumin, (g/L)	44 (6)	44 (5)	44 (4)	0.38
Plasma potassium, (mmol/L)	4.2 (0.2)	4.2 (0.3)	4.2 (0.3)	0.002
Plasma sodium, (mmol/L)	141 (2)	141 (2)	141 (2)	0.12
High sensitive CRP, (mg/L)	1.0 (0.5 – 2.4)	1.1 (0.5 – 2.4)	1.2 (0.5 – 2.7)	0.05
Plasma phosphate, (mmol/L)	1.00 (0.33)	1.03 (0.34)	1.05 (0.46)	0.005
Plasma PTH, (pmol/L)	4.7 (3.9 – 5.5)	4.7 (4.0 – 5.7)	4.8 (4.0 – 5.8)	0.006
Plasma vitamin D_3, 25-OH, (nmol/L)	58.7 (41.2 – 77.3)	58.7 (42.6 – 78.0)	54.2 (37.9 – 74.4)	<0.001
Plasma calcium, (mmol/L)	2.28 (0.11)	2.30 (0.11)	2.30 (0.12)	<0.001
Plasma calcium corrected, (mmol/L)	2.20 (0.16)	2.22 (0.14)	2.22 (0.16)	0.001
Iron, (umol/L)	17 (6)	17 (6)	15 (6)	<0.001
Ferritin, (ug/L)	92 (50 – 159)	88 (45 – 156)	64 (25 – 131)	<0.001
Transferrin saturation, (%)	26 (9)	26 (9)	22 (10)	<0.001
Urinary sodium excretion, (mmol/24hrs)	141 (109 – 174)	138 (104 – 174)	136 (104 – 172)	0.07
Urinary potassium excretion, (mmol/24hrs)	70 (23)	70 (22)	68 (22)	0.006
Urinary phosphate excretion, (mmol/24hrs)	25 (19 – 32)	26 (20 – 33)	25 (19 – 32)	0.27
TMP/GFR	0.97 (0.80 – 1.19)	1.01 (0.82 – 1.23)	1.03 (0.83 – 1.23)	0.002
Urinary creatinine excretion, (mmol/24hrs)	13 (3)	12 (3)	12 (3)	0.10

Urinary albumin excretion, (mg/24hrs)	11.6 (8.8 – 18.3)	11.3 (8.5 – 17.1)	11.8 (8.7 – 19.3)	0.06
Urinary albumin-to creatinine ratio, (mg/mmol)	0.64 (0.48 – 1.03)	0.63 (0.47 – 0.98)	0.67 (0.48 – 1.08)	0.05

Data are presented as n (%), mean (SD), or median [interquartile range] for nominal, normally distributed, and non-normally distributed data, respectively.

*The *P*-value represents the p for trend in Chi-square test, one-way-ANOVA, or Kruskal Wallis test for nominal, normally distributed and non-homogeneity or non-normally distributed data, respectively.

Abbreviations: BMI, Body Mass Index; eGFR (CKD-epi), estimated glomerular filtration rate based on Chronic Kidney Disease Epidemiology Collaboration equation; CRP, C-reactive protein; PTH, parathyroid hormone; TMP/GFR, renal tubular reabsorption of phosphate.

Supplemental Table S2. Multivariable associations of urinary potassium excretion and fibroblast growth factor 23 with systolic and diastolic blood pressure in 4,194 participants of the PREVEND cohort

	Systolic blood pressure		Diastolic blood pressure	
	Standardized Beta	*P*-value	Standardized Beta	*P*-value
Urinary potassium excretion, mmol/24hrs*	0.02	0.24	−0.02	0.16
(Log_2)FGF23, RU/mL*	0.005	0.35	0.001	0.93

* Models were multivariable adjusted for age, BMI, eGFR, urinary sodium excretion, urinary albumin-to-creatinine ratio, smoking status, alcohol intake, diabetes and cardiovascular disease. Abbreviations: BMI, Body Mass Index; eGFR, estimated glomerular filtration rate; FGF23; Fibroblast growth factor 23.
